# Identifying HER2 Inhibitors from Natural Products Database

**DOI:** 10.1371/journal.pone.0028793

**Published:** 2011-12-12

**Authors:** Shun-Chieh Yang, Su-Sen Chang, Calvin Yu-Chian Chen

**Affiliations:** 1 Laboratory of Computational and Systems Biology, China Medical University, Taichung, Taiwan; 2 Department of Bioinformatics, Asia University, Taichung, Taiwan; 3 China Medical University Beigang Hospital, Yunlin, Taiwan; 4 Department of Systems Biology, Harvard Medical School, Boston, Massachusetts, United States of America; 5 Computational and Systems Biology, Massachusetts Institute of Technology, Cambridge, Massachusetts, United States of America; Kings College, London, United Kingdom

## Abstract

The relationship between abnormal HER2 expression and cancer is important in cancer therapeutics. Formation and spread of cancer cells may be restricted by inhibiting HER2. We conducted ligand-based and structure-based studies to assess the potency of natural compounds as potential HER2 inhibitors. Multiple linear regression (MLR) and support vector machine (SVM) models were constructed to predict biological activities of natural compounds, and molecular dynamics (MD) was used to assess their stability with HER2 under a dynamic environment. Predicted bioactivities of the natural compounds ranged from 6.014–9.077 using MLR (r^2^ = 0.7954) and 5.122–6.950 using SVM (r^2^ = 0.8620). Both models were in agreement and suggest bioactivity based on candidate structure. Conformation changes caused by MD favored the formation of stabilizing H-bonds. All candidates had higher stability than Lapinatib, which may be due to the number and spatial distribution of additional H-bonds and hydrophobic interactions. Amino acids Lys724 and Lys736 are critical for binding in HER2, and Thr798, Cys805, and Asp808 are also important for increased stability. Candidates may block the entrance to the ATP binding site located within the inner regions and prevent downstream activation of HER2. Our multidirectional approach indicates that the natural compounds have good ligand efficacy in addition to stable binding affinities to HER2, and should be potent candidates of HER2 inhibitors. With regard to drug design, designing HER2 inhibitors with carboxyl or carbonyl groups available for H-bond formation with Lys724 and Lys736, and benzene groups for hydrophobic contact with Cys805 may improve protein-ligand stability.

## Introduction

HER2 are members of the epidermal growth factor receptor tyrosine kinase protein family which includes HER1/EGFR, HER2/ErbB2, HER3/ErbB3, and ErbB4. These proteins form various homo- and hetero- dimer receptors on human cell membranes. When these receptors bind with ligands, autophosphorylation will occur and activate P13k/Akt and Ras/Raf signaling pathways, stimulating signal transduction of downstream cell growth and differentiation [1], [2]. Clinically, abnormalities in HER2 gene regulation will cause receptor over-production, resulting in various cancers including breast cancer, ovarian cancer, gastric cancer, and prostate cancer [3]–[7]. Therefore, inhibiting HER2 expression and function is critical in treating cancer and preventing the spread of cancerous cells.

Trastuzumab (Herceptin®) and Lapatinib (Tykerb®) are two drugs used clinically in breast cancer. Trastuzumab inhibits over-expression of HER2 [8], and Lapatinib inhibits HER2 autophosphorylation by competing with ATP for the HER2 protein kinase domain, thus preventing further signal transduction [9]. Drug resistance issues have been reported for Trastuzumab [10]. Synergistic effects on breast cancer is observed when Lapatinib is used with Capecitabine, but side effects such as nausea, vomiting, and diarrhea have been recorded [11].

Computer-aided drug design is widely used in developing new drugs and has been integrated in this laboratory with our self-developed TCM Database@Taiwan [12] to design and develop novel drugs from traditional Chinese medicine [13]–[17]. Much research has proven that traditional Chinese herb compounds exhibit antioxidation and anti-inflammation effects and have therapeutic effects on cancer [18]–[20]. A preliminary experiment conducted in this laboratory identified several natural compounds from traditional Chinese herbs as HER2 inhibitors through docking and 3D-QSAR evaluation [21]. However, as static state docking does not necessarily equal stability in a dynamic state (ie. body), further evaluation is required. This research aims to predict biological activity with different statistical models, and evaluate candidate-HER2 complex stability under a dynamic state.

## Materials and Methods

### Candidate Compounds and Docking Site

Based on our previous findings [21], natural compounds 2-O-caffeoyl tartaric acid, 2-O-feruloyl tartaric acid, and salvianolic acid C exhibited good docking characteristics and were selected as candidates for further investigation. Lapatinib was used as the control. The HER2 docking site was constructed through sequence homology and detailed elsewhere [21].

### Biological Activity Prediction using Multiple Linear Regression (MLR) and Support Vector Machine (SVM) Models

A total of 298 HER2 ligands were adapted to construct activity (pIC_50_) prediction models [22]–[35]. Descriptors of each ligand were calculated using the Calculate Molecular Properties module in Discovery Studio 2.5 (DS 2.5; Accelrys, San Diego, CA) and plugged into the Genetic Approximation (GA) algorithm to select 12 optimum descriptors for predicting pIC_50_. The selected descriptors were used to construct MLR and SVM models using Matlab Statistics Toolbox and libSVM, respectively. Descriptors were normalized between [−1,+1] before SVM model training. Gaussian radial basis function was selected as the kernel function for SVM model generation. The HER2 ligands were randomly divided into a 238 ligand training set and a 60 ligand test set for validation. Prediction results were validated with 5-fold cross validation. The constructed models were applied to predict biological activities (pIC_50_) of the control and top 3 natural compounds.

### Molecular Dynamics (MD) Simulation

The HER2 protein structure used within this study was constructed through homology modeling using EGFR kinase domain structures found in Protein Data Bank (PDB: 2ITY and 2J5E). Modeling details and validity testing are detailed in our previous study [21]. Molecular dynamics simulation was carried out using DS 2.5 Standard Dynamics Cascade package with the following settings: [minimization] steepest descent, [conjugate gradient] maximum steps of 500, [heating time] 50 ps, [equilibration time] 200 ps, [total production time] 20 ns with NVT, [constant temperature dynamics] Berendsen weak coupling method, [temperature coupling decay time] 0.4 ps with the Berendsen thermal coupling method, and [target temperature] 310 K. Hydrogen bonds, distance of hydrogen bond, root mean square deviations (RMSD) of complex, RMSD of ligand, total energy of complex, and torsion angles were analyzed by the analyze trajectory protocol of DS 2.5 following MD simulation. Protein-ligand interactions were analyzed with the LIGPLOT program [36].

## Results and Discussion

### Biological Activity Predictions using MLR and SVM

**Figure 1:**
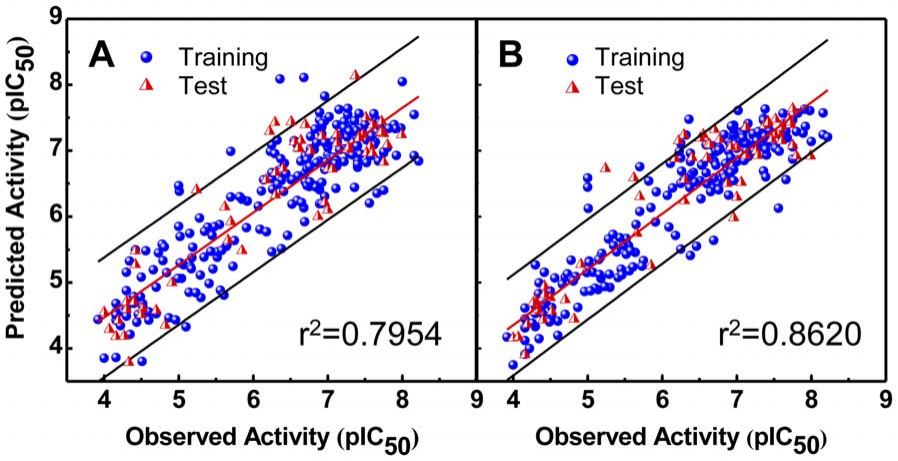
Correlation of observed and predicted activity (pIC_50_) by different prediction models. (A) MLR and (B) SVM.

The following MLR model was generated utilizing the GA descriptors and 238 training-set ligands:
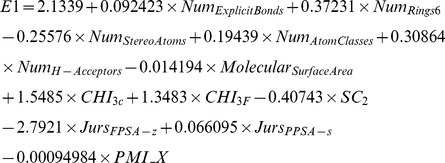
Correlation of actual and predicted activities of HER2 ligands based on the generated MLR are shown in Figure 1A and the residual plot indicating the goodness-of-fit is shown in Figure S1. Majority of the predicted values are within the 95% prediction bands, indicating acceptable correlation of the MLR model (r^2^ = 0.7954). Similarly, the SVM model was generated using identical GA descriptors and the results are illustrated in Figure 1B. Acceptable correlation was also observed (r^2^ = 0.8620). Based on these results, the models are acceptable models for predicting activity of HER2 ligands.

Table 1 summarizes the predicted biological activities for Lapatinib and the candidate compounds using MLR and SVM models. All compounds were predicted as biologically active and are in agreement with 3D-QSAR results previously reported [21]. In contrast to descriptive bioactivity predictions based on 3D-QSAR results, MLR and SVM predictions allow quantitative predictions on the strength of bioactivity in each compound. Salvianolic acid C showed the highest bioactivity among TCM candidates.

**Table 1:** DockScore and predicted activities of candidate compounds using MLR and SVM.

Compounds	Dock Score^a^	MLR	SVM
2-O-Caffeoyl tartaric acid	121.870	6.879	5.339
2-O-Feruloyl tartaric acid	121.483	6.014	5.122
Salvianolic acid C	104.833	9.077	6.950
Lapatinib*	67.330	7.640	7.058

a : scores adapted from Sun et al. [21].

* : control.

### Molecular Dynamics Simulation

**Figure 2:**
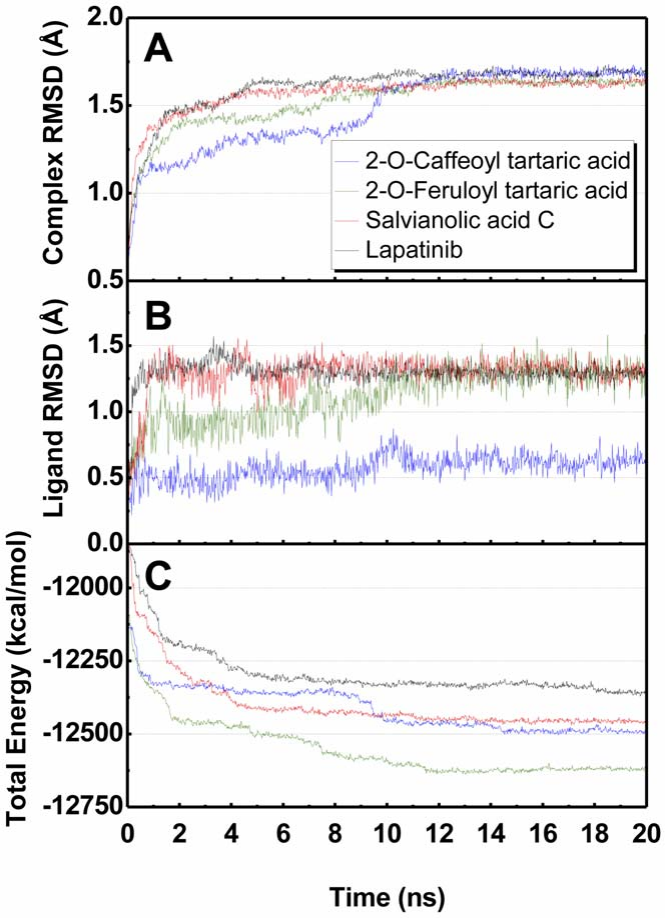
Trajectory profiles of RMSD and total energy during molecular dynamics simulation. (A) Complex RMSD, (B) ligand RMSD, and (C) total energy.

Stability of Lapatinib and the TCM candidates were achieved after 10 ns in a dynamic environment (Figure 2). The RMSD of whole complexes was approximately 1.6 Å (Figure 2A). The smallest RMSD was observed in 2-O-caffeoyl tartaric acid (0.6 Å). The RMSD of the other compounds were *ca.* 1.3 Å (Figure 2B). The small variation of 2-O-caffeoyl tartaric acid suggests a stable state within the receptor site. Total energy trajectories indicate that the candidates form complexes with lower energy compared to the control. The higher stability further supports the potential of the candidates as drug alternatives to Lapatinib.

**Figure 3:**
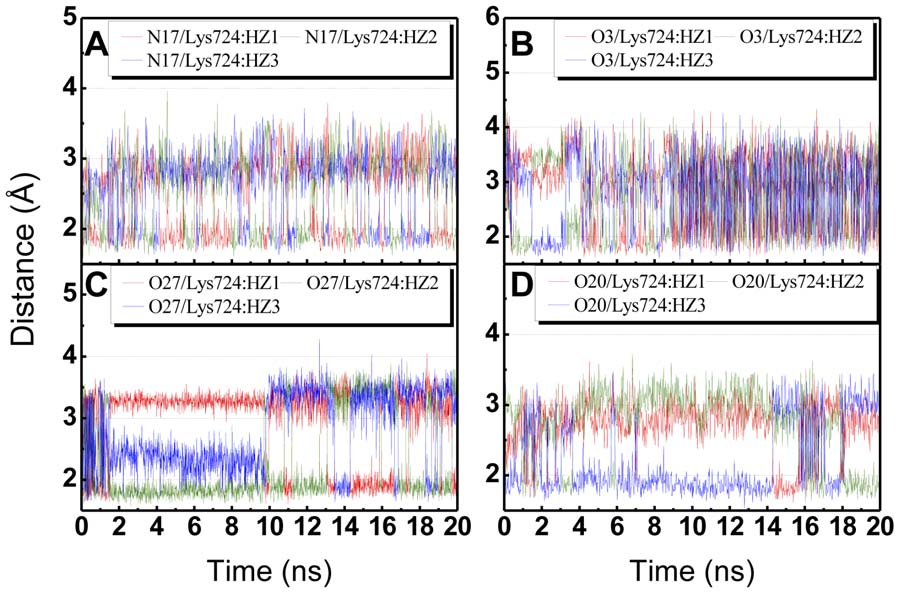
Hydrogen bond distance (Å) of candidates during MD simulation. (A) Lapatinib, (B) 2-O-Caffeoyl tartaric acid, (C) 2-O-Feruloyl tartaric acid, and (D) Salvianolic acid C.

The amine residue on Lys724 in HER2 protein is important to form hydrogen bonds (H-bond) with ligands. Each ligand formed at least one stable H-bond with Lys724 throughout the MD simulation (Figure 3). The amine residue constantly rotates when bound to Lapatinib. Overlapping of the indicator lines suggests the existence of multiple H-bonds at Lys724 with 2-O-caffeoyl tartaric acid after 10 ns. H-bonds with 2-O-feruloyl tartaric acid and salvianolic acid C were fairly stable. Rotation was observed with salvianolic acid C from 14–20 ns.

**Figure 4:**
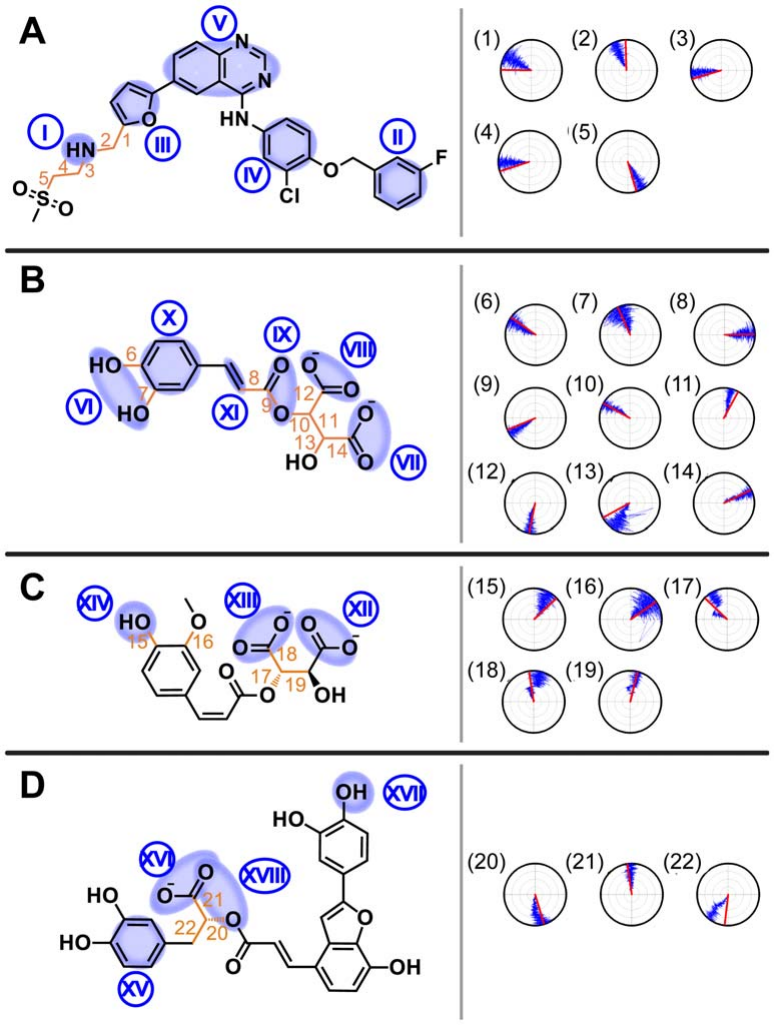
Structural scaffolds of candidate compounds. (A) Lapatinib, (B) 2-O-Caffeoyl tartaric acid, (C) 2-O-Feruloyl tartaric acid, and (D) Salvianolic acid C. Locations of importance during MD are denoted by numbers.

**Figure 5:**
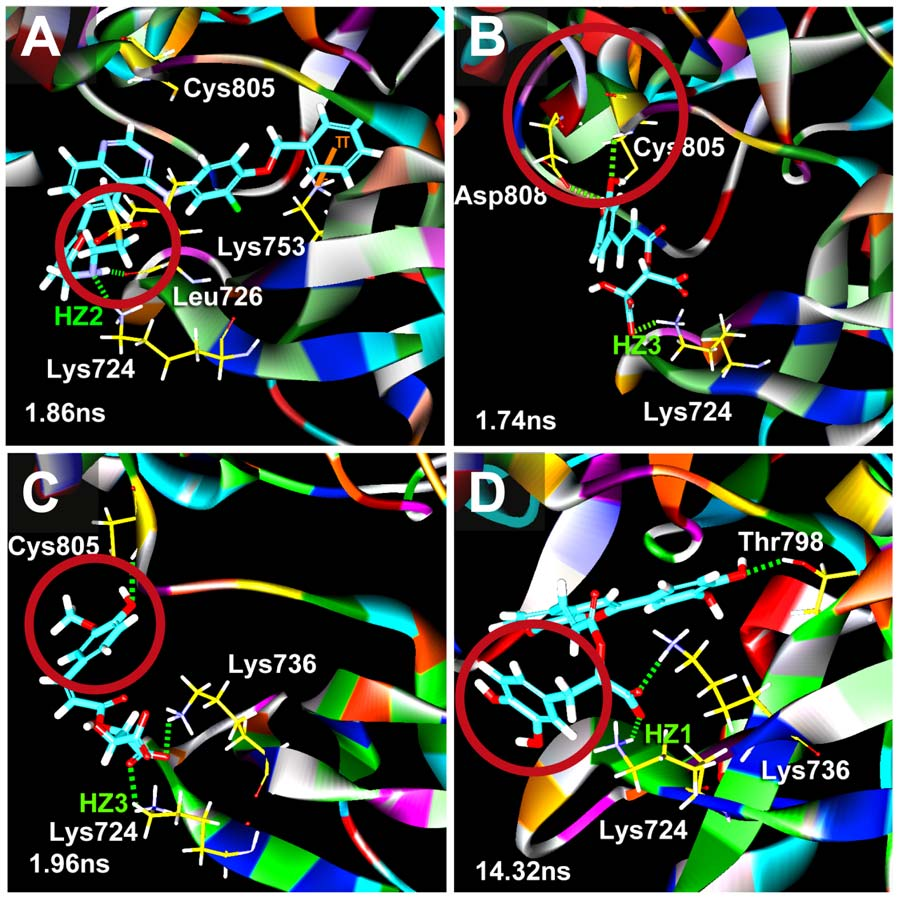
Binding pose of candidate compounds in HER2. (A) Lapatinib, (B) 2-O-Caffeoyl tartaric acid, (C) 2-O-Feruloyl tartaric acid, and (D) Salvianolic acid C. Differences from the docking pose are specified by the red circle. Hydrogen-bond interactions (green dashed line), Pi interactions (orange line). The three H atoms of Lys 724 amine subgroup are labeled as HZ1, HZ2, and HZ3.

Stability mechanisms of each candidate can be provided by MD simulation trajectories. For clarification purposes, specific locations are denoted by Roman numerals and torsion angle locations are designated by Arabic numerals in Figure 4. In Lapatinib, the bond between Lys724 and the SO_2_ residue in docking was substituted by the NH group (red circle) at the beginning of MD (Figure 5A, S1A). The shift occurred because the bond between NH_3_^+^-N is stronger than that of NH_3_^+^-SO_2_, and might have been triggered by procedures (minimization, heating, and equilibration) prior to MD production. During MD, Lapatinib formed stable H-bonds with Lys724 and Leu726 at **I**, and a stable pi-cation interaction with Lys753 at II (Figure 4A). Torsion angles indicate bonds **1–5** were stable, most likely due to the H-bond at **I**. Moieties **II** and **III** were also important to the stability of Lapatinib. At approximately 0.16 ns, **II** rotated to being perpendicular to **IV**. The conformation change may have contributed to the decrease in total energy by reducing strain on the connecting O atom. The rotation of **III** to being perpendicular to **V** also coincided with the reduction of total energy in Figure 2C.

Differences from docking were also observed for 2-O-caffeoyl tartaric acid. Cys805-Asp808 formed a helix structure during docking (Figure S2B). During MD, a fold in the original helix (red circle, Figure 5B) enabled formation of stable H-bonds between Asp808 and **VI**. In addition, 2-O-caffeoyl tartaric acid was anchored to the binding site by multiple H-bonds formed through **VII** and **VIII** with Lys724, **VIII** and **IX** with Lys736, and **X** with Cys805. Similar to being locked-down, movement of the ligand was limited except at **XI**. Additional evidence is provided by the small torsion angles recorded for the backbone structure of 2-O-caffeoyl tartaric acid. Changes in torsion were generally small, and notable movement was only observed on hydroxyl side residues **6**, **7**, **13** (Figure 4B). These observations can explain the low ligand RMSD (Figure 2B) and support the stability of the formed complex.

A *ca.* 180 degree rotation of the benzene on 2-O-feruloyl tartaric acid was observed during MD (Figure 5C, S1C). The rotation flipped **XII** towards Lys736, and H-bonds were formed. Additional H-bonds were formed at **XIII** with Lys724 and at **XIV** with Cys805. All formed H-bonds were stable throughout MD. Small torsion changes were observed for 2-O-feruloyl tartaric acid except at **16** which was not restricted by any bond formation (Figure 4C). The compact structure and the even distribution of the H-bond formation locations may be the reason 2-O-feruloyl tartaric acid has the highest stability (ie. lowest total energy) among all the tested compounds (Figure 2C).

Conformational changes contributing to higher stability was also observed in salvianolic acid C (Figure 5D, Figure S2D). The benzene structure **XV** on salvianolic acid C torqued nearly 90 degrees during MD (red circle, Figure 5D), and the pi-interaction originally observed during docking was lost. The rotation relocated available residues to favorable H-bond forming locations, and H-bonds were formed between **XVI** and Lys736 and **XVII** and Thr798. The pi-cation interaction with Lys724 was replaced by the H-bonds formed at **XVIII** with the three available H atoms from Lys724. Small torsion angles (Figure 4D) provide evidence of the ligand stability.

**Figure 6:**
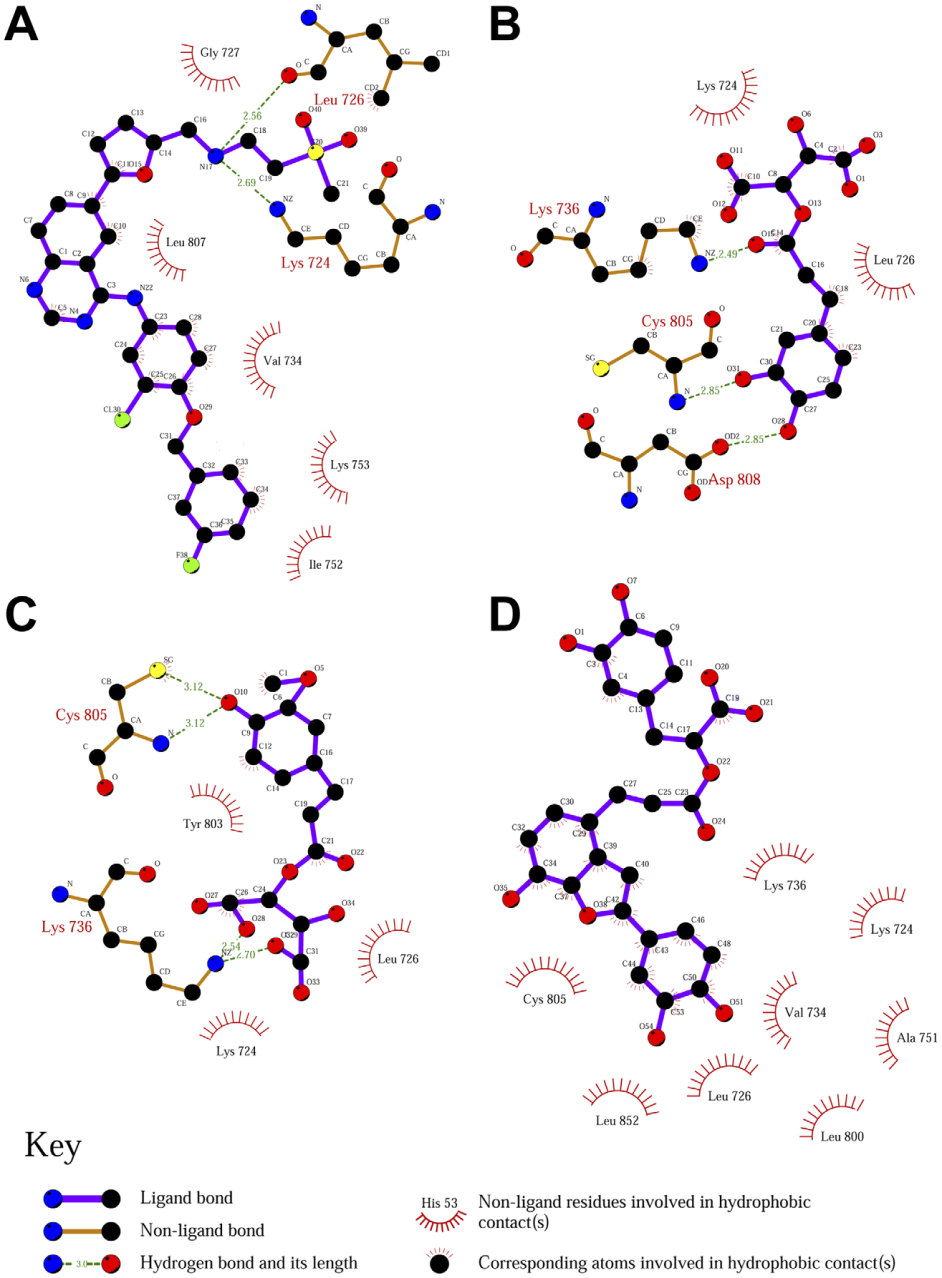
Protein-ligand interaction analysis by LIGPLOT. (A) Lapatinib, (B) 2-O-Caffeoyl tartaric acid, (C) 2-O-Feruloyl tartaric acid, and (D) Salvianolic acid C.

Hydrophobic interactions determined for each ligand at the end of MD are illustrated in Figure 6. In addition to the previously discussed H-bonds, Lapatinib was further stabilized through hydrophobic interaction with Gly727, Val734, Ile752, Lys753, and Leu807 (Figure 6A). Lys724 and Leu726 were important in forming hydrophobic interactions with 2-O-caffeoyl tartaric acid (Figure 6B) and 2-O-feruloyl tartaric acid (Figure 6C). An additional hydrophobic interaction with Tyr803 was observed in 2-O-feruloyl tartaric acid. Eight amino acids were detected as exhibiting hydrophobic interaction on Salvianolic acid C (Figure 6D). Majority of the cyclic carbon moieties were stabilized through these interactions.

**Figure 7:**
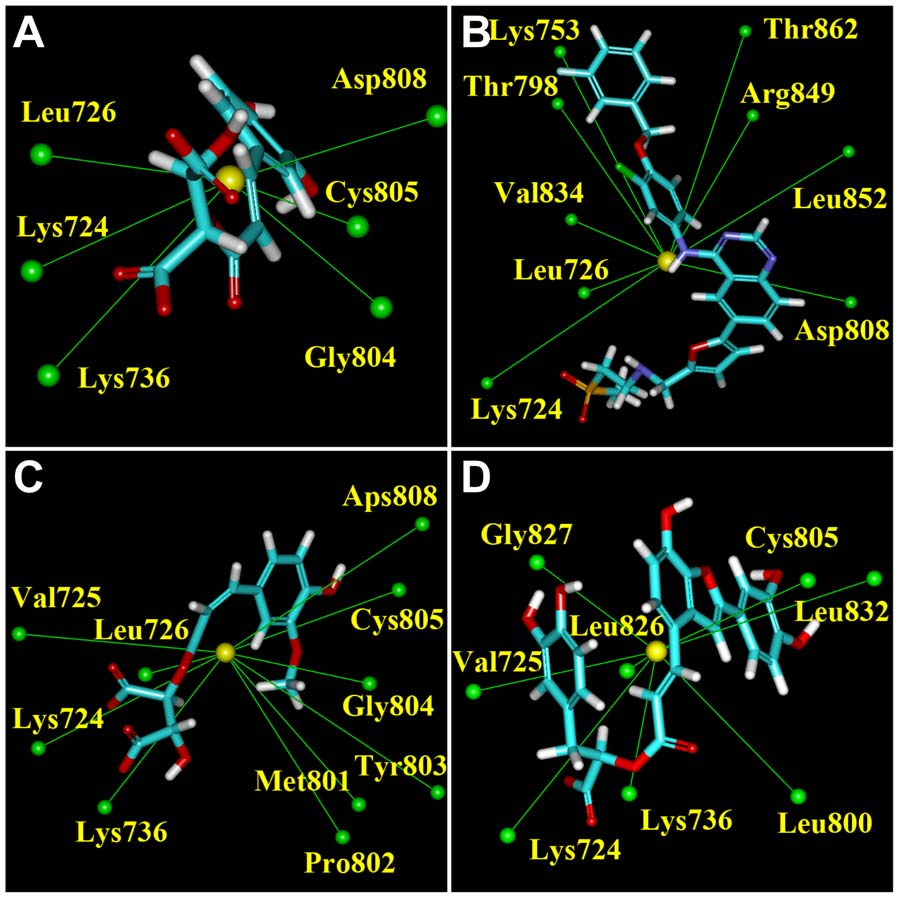
Centroid distance between candidates and proximate HER2 amino acids. (A) Lapatinib, (B) 2-O-Caffeoyl tartaric acid, (C) 2-O-Feruloyl tartaric acid, and (D) Salvianolic acid C. Centroids of the ligands and amino acids are represented in yellow and green, respectively.

**Figure 8:**
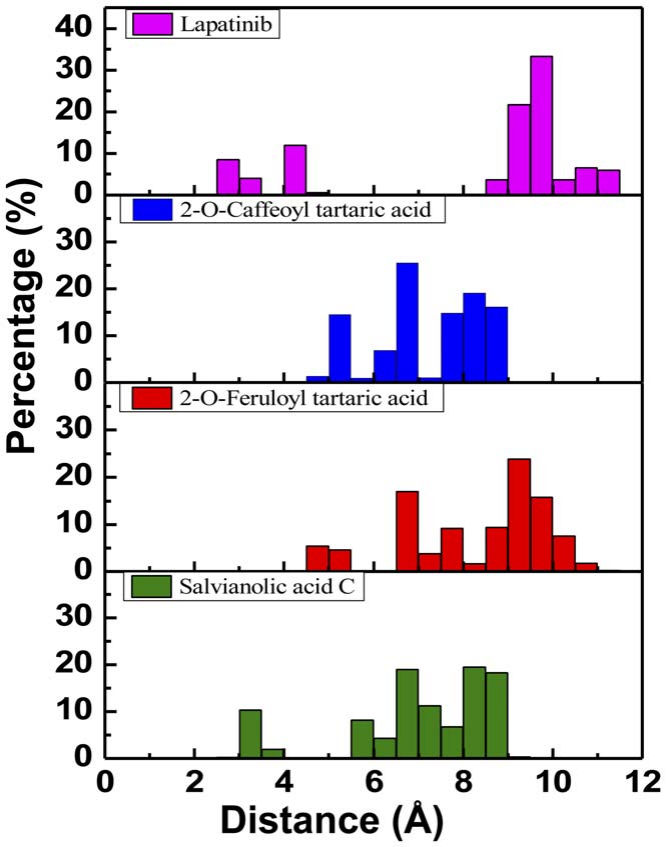
Distribution of centroid distance (Å) between ligand and proximate residues.

**Figure 9:**
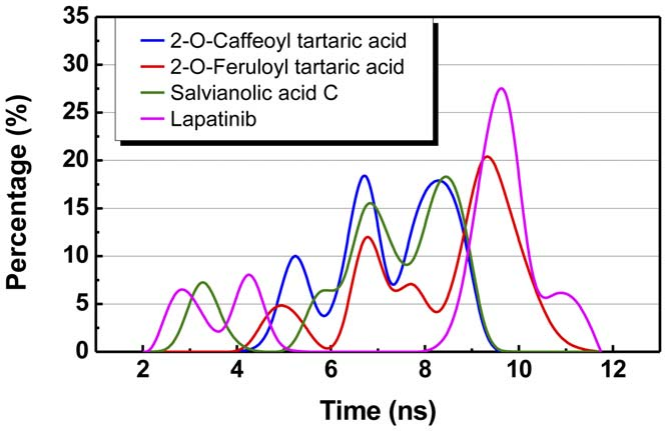
Population of centroid distance (Å) between ligand and proximate residues.

The spatial location and distances of nearby amino acids with the centroid of each candidate ligand are depicted in Figures 7 and 8. A bimodal distribution of amino acid distances was observed for Lapatinib. On the other hand, the distance of nearby amino acids from the centroid of the TCM candidates were more uniform. The distance distribution (Figure 9) suggests that all test ligands were tightly fitted within the binding site and can effectively block ATP from binding. Furthermore, the candidates were more closely bound to the binding site than Lapatinib, indicating another advantage of the candidates as a potential Lapatinib substitute.

MD observations indicate that the candidate compounds are more stable within the HER2 binding site than Lapatinib. The stability could be explained in part by the multiple H-bonds formed with the binding site. Conformational changes induced by the MD simulation were favorable in forming additional H-bonds that contributed to overall stability of the candidates.

**Figure 10:**
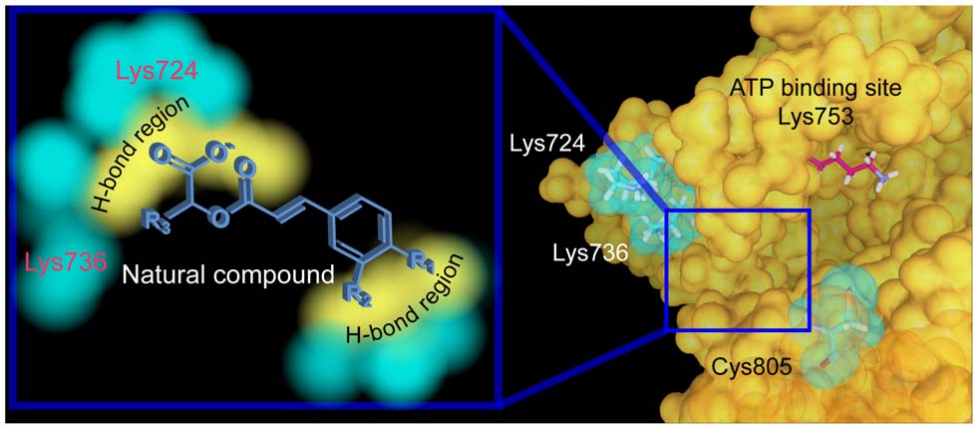
Summary of critical features for HER2 inhibition and possible inhibition mechanism.

Possibility of the natural compound candidates as alternatives to Lapatinib was supported by the ligand based analysis and MD simulation. Candidates were predicted as biologically active by the constructed MLR (r^2^ = 0.7954) and SVM (r^2^ = 0.8620) models based on their ligand characteristics. Molecular simulation revealed that candidates formed more stable complexes with the HER2 binding site (*ie.* lower in total energy) than Lapatinib. This increased stability may be explained by the formation of additional stabilizing H-bonds and hydrophobic contacts. Figure 10 summarizes the key conclusions from the preliminary study [21] and this current investigation. Amino acids that are critical for HER2-ligand interaction include Lys724, Lys736, and Cys805. As illustrated in Figure 10, binding at the key amino acids results in blocking of the ATP binding site entrance, and may result in inhibition of HER2 activity. Analysis of the candidates indicates that carbonyl, carboxylic acid, and hydroxyl groups are critical moieties for stable binding. Based on the results of this study, the natural compound candidates have potential as biologically active compounds with improved stability in HER2. Designing HER2 inhibitors with carbonyl, carboxyl, and hydroxyl groups available for H-bond formation may improve protein-ligand stability.

## Supporting Information

Figure S1 **Residual plot indicating the goodness-of-fit for the constructed MLR model.**
(TIF)

Figure S2 **Docking pose of TCM candidates in HER2.** (A) Lapatinib, (B) 2-O-Caffeoyl tartaric acid, (C) 2-O-Feruloyl tartaric acid, and (D) Salvianolic acid C. Green dashed lines and orange lines refer to H-bonds and π-interactions, respectively, Illustration adapted from Sun et al. [21] with the permission of the authors.
(TIF)
