# Supplementary figures and images for: Identifying HER2 Inhibitors from Natural Products Database
Source: PLoS One. 2011 Dec 12;6(12):e28793. doi: 10.1371/journal.pone.0028793 (PMC3236219; doi:10.1371/journal.pone.0028793)

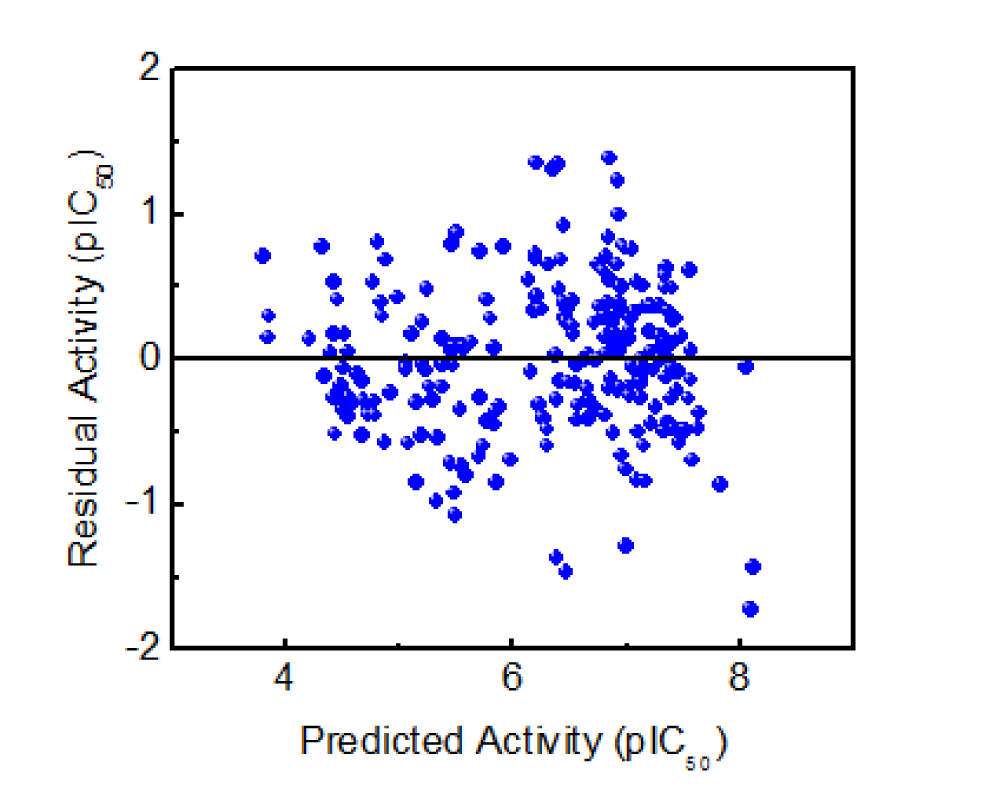

Supplement: Figure S1 — Residual plot indicating the goodness-of-fit for the constructed MLR model. (TIF) [file pone.0028793.s001.tif]

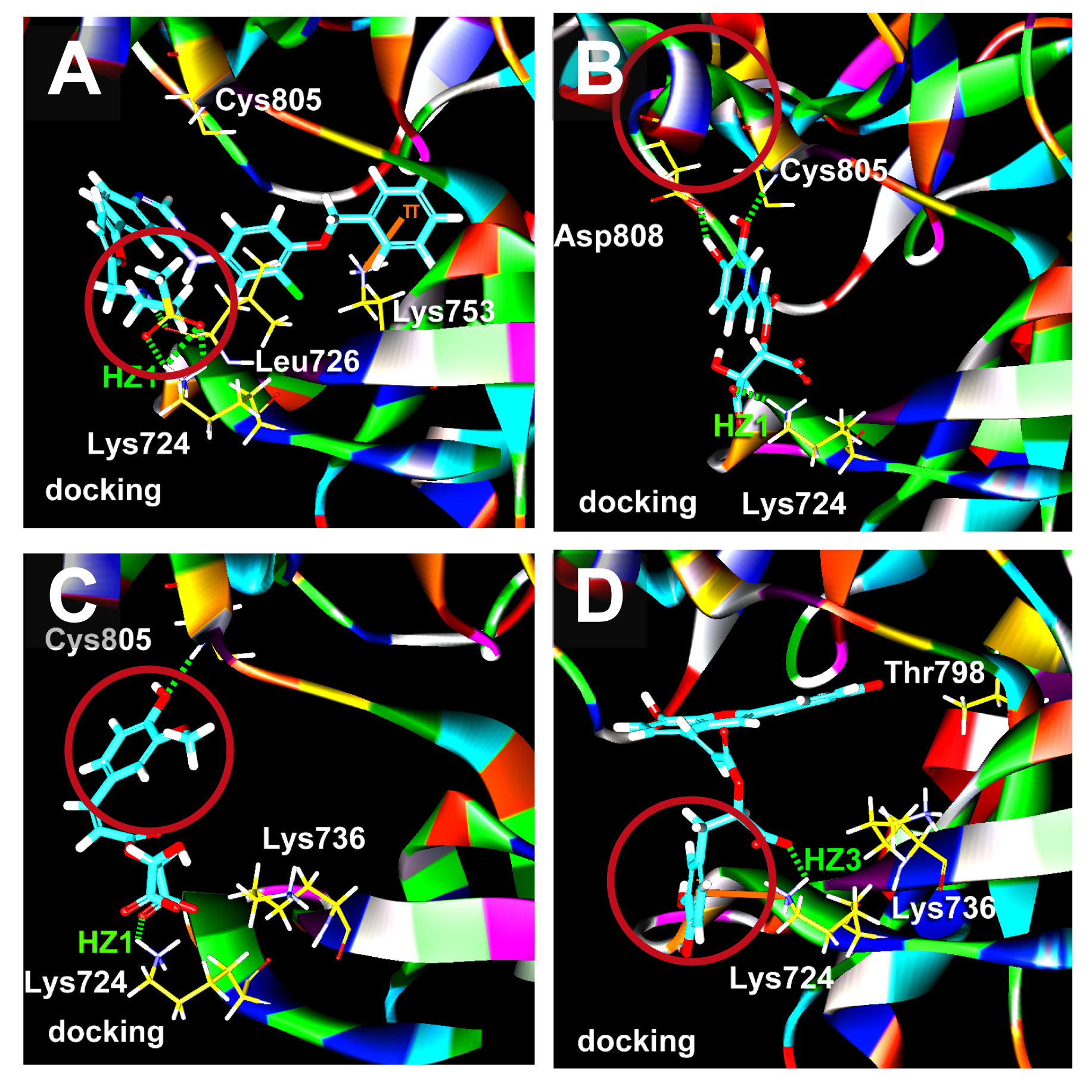

Supplement: Figure S2 — Docking pose of TCM candidates in HER2. (A) Lapatinib, (B) 2-O-Caffeoyl tartaric acid, (C) 2-O-Feruloyl tartaric acid, and (D) Salvianolic acid C. Green dashed lines and orange lines refer to H-bonds and π-interactions, respectively, Illustration adapted from Sun et al. [21] with the permission of the authors. (TIF) [file pone.0028793.s002.tif]
